# Adsorption and Conformation Behavior of Lysozyme on a Gold Surface Determined by QCM-D, MP-SPR, and FTIR

**DOI:** 10.3390/ijms22031322

**Published:** 2021-01-28

**Authors:** Paulina Komorek, Elisha Martin, Barbara Jachimska

**Affiliations:** 1 Jerzy Haber Institute of Catalysis and Surface Chemistry Polish Academy of Sciences, Niezapominajek 8, 30-239 Cracow, Poland; paulina.komorek@ikifp.edu.pl; 2 Department of Chemical and Process Engineering, University of Strathclyde, 75 Montrose Street, Glasgow G1 1XJ, UK; elisha.martin@strath.ac.uk

**Keywords:** lysozyme adsorption, gold surface, hydration, viscoelastic properties, conformation, QCM-D, MP-SPR, FTIR

## Abstract

The physicochemical properties of protein layers at the solid–liquid interface are essential in many biological processes. This study aimed to link the structural analysis of adsorbed lysozyme at the water/gold surface at pH 7.5 in a wide range of concentrations. Particular attention was paid to the protein’s structural stability and the hydration of the protein layers formed at the interface. Complementary methods such as multi-parameter surface plasmon resonance (MP-SPR), quartz crystal microbalance with energy dissipation (QCM-D), and infrared spectroscopy (FTIR) were used for this purpose. The MP-SPR and QCM-D studies showed that, during the formation of a monolayer on the gold surface, the molecules’ orientation changes from side-on to end-on. In addition, bilayer formation is observed when adsorbing in the high-volume concentration range >500 ppm. The degree of hydration of the monolayer and bilayer varies depending on the degree of surface coverage. The hydration of the system decreases with filling the layer in both the monolayer and the bilayer. Hydration for the monolayer varies in the range of 50–70%, because the bilayer is much higher than 80%. The degree of hydration of the adsorption layer has a crucial influence on the protein layers’ viscoelastic properties. In general, an increase in the filling of a layer is characterized by a rise in its rigidity. The use of infrared spectroscopy allowed us to determine the changes taking place in the secondary structure of lysozyme due to its interaction with the gold surface. Upon adsorption, the content of II-structures corresponding to β-turn and random lysozyme structures increases, with a simultaneous decrease in the content of the β-sheet. The increase in the range of β-turn in the structure determines the lysozyme structure’s stability and prevents its aggregation.

## 1. Introduction

The interaction of proteins with solid surfaces is a crucial phenomenon, influencing many biological and industrial processes. For instance, adsorption of blood proteins on some materials not adapted to biomedical applications can lead to thrombosis [1,2], in the chromatography technique, it provides the opportunity for phases separation [3,4], while in the biotechnological area, it can act as the basis for designing biosensors [5,6,7,8]. It was also noticed that interactions between proteins and surfaces could induce the formation or stabilization of structures characteristic for neurodegenerative diseases [9,10,11]. Therefore, control of the stability of protein structures has great potential for their use in neurological diagnostics. Most of the processes require controlling adsorption effectiveness (its maximization or minimization); therefore, many studies focus on developing appropriate quantitative adsorption models [12,13,14,15]. Although the proposed models correlate well with the results of the adsorption efficiency study, they do not allow the properties of the resulting layers to be determined. It should be noted that the properties of the resulting protein layers are crucial for technological reasons. This is because all proteins’ functions and activity depend on their biological and chemical parameters, which are also directly related to their structure [16,17]. For this reason, multi-faceted characterization of proteins after their adsorption can contribute to the understanding of structure-property relationships, which is so essential in the application area [18,19,20,21,22,23]. So far, many experiments have been carried out on macroscopic properties, such as the hydrophobicity [24,25] and the topography of the formed layers. Due to biomedical applications, the hydration of protein layers is particularly important, but is not well understood. This aspect was studied, among other things, by Ouberai et al., who determined the hydration of lysozymes depending on the surface coverage [26]. The literature includes several papers on the viscoelastic properties of the adsorbed protein layers [27,28], with one study, in particular, proving especially interesting, determining the flexibility for proteins that differ in their native structure [26]. There are also investigations focused on protein secondary structure changes after adsorption, which are crucial for maintaining biological activity [29,30,31,32,33]. However, there is still a lack of comprehensive research combining studies on the effectiveness of adsorption and hydration of formed films and their viscoelastic properties with the influence on the adsorbed protein’s conformation. In the presented work, we selected a hen egg lysozyme and a gold surface to study the effects of adsorption carried out in different conditions on formed layers’ properties. The measurements were performed depending on the bulk protein concentration for two different ionic strengths (I = 0.01 M and I = 0.15 M NaCl). The effectiveness of adsorption was established by multi-parametric surface plasmon resonance (MP-SPR) and quartz crystal microbalance with dissipation monitoring (QCM-D). The results, combined with random sequential adsorption model for ellipsoidal shape molecules, were also used to determine the orientation of molecules and structure (monolayer/bilayer) of adsorbed films. Comparison of MP-SPR and QCM-D data enabled the hydration of protein layers to be calculated, while using frequency and dissipation QCM-D results in the elasticity and layer shear viscosity being determined. Overall, this comprehensive study presents conclusions about the properties of lysozyme layers due to protein adsorption. They can be crucial for protein application both in biotechnology and nanotechnology.

## 2. Results and Discussion

### 2.1. Adsorption of Lysozymes on Gold Surface Monitored by Multi-Parametric Surface Plasmon Resonance Method (MP-SPR)

**Figure 1:**
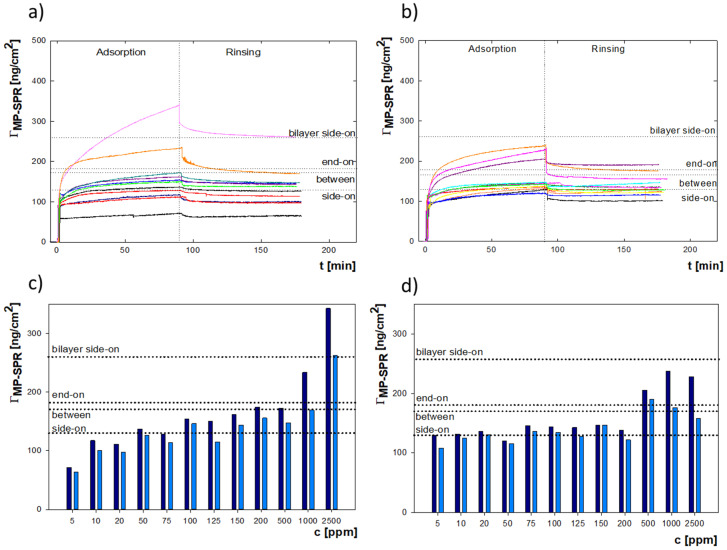
Time dependence of the mass of adsorbed lysozyme (*Г_MP-SPR_*) on the gold surface for concentration range of 5–2500 ppm monitored by MP-SPR at (**a**) I = 0.01 M; (**b**) I = 0.15 M. Adsorbed lysozyme mass on the gold surface after 90 min of adsorption (navy blue) and after another 90 min of rinsing with NaCl (blue) at (**c**) I = 0.01 M; and (**d**) I = 0.15 M at pH = 7.5. The dashed lines indicate the masses corresponding to the particular orientations of the molecules.

The adsorption of lysozymes on the gold surface was examined using the MP-SPR method. The measurements were performed depending on the bulk protein solution’s concentration for two ionic strengths: 0.01 M and 0.15 M NaCl at pH = 7.5. According to previous results, the isoelectric points of lysozyme and the gold surface are pH = 10 and pH = 3.4 [30,34]. At the selected pH = 7.5, the zeta potential of gold is about −20 mV, and that of lysozyme is equal to 7 mV. Therefore, there are attractive electrostatic interactions between positively charged lysozyme particles and a negatively charged surface [30,34]. In the MP-SPR method, the changes of resonance angle for specified concentrations were converted into changes in the mass adsorbed on the sensor. The obtained results are shown in Figure 1a,b. According to the calculations based on the random sequential adsorption model for ellipsoidal particles [34,35], the monolayer in the side-on orientation corresponds to an adsorbed mass of 129.38 ng/cm^2^, between—mass of 176.91 ng/cm^2^, and end-on—mass of 183.71 ng/cm^2^ [30]. Figure 1c shows the results for the ionic strength equal to 0.01 M. On its basis, it can be concluded that for lysozyme concentrations in the range of 5–75 ppm, a monolayer is formed in the side-on orientation; at 100 ppm, a full monolayer is obtained. Then, in the concentration range between 100 and 500 ppm, the molecules’ reorientation between orientation and the complete end-on monolayer occurs. Increasing the concentration above 500 ppm results in forming a side-on bilayer, which is achieved at 2500 ppm and remains stable even after rinsing with 0.01 M NaCl. For measurements performed at I = 0.15 M (Figure 1d) in the low concentration range, there is higher efficiency of adsorption than for experiments carried out at I = 0.01 M. At 10 ppm, a full monolayer in side-on orientation is formed; a ten-fold rise in concentration does not cause significant changes. In comparison, at a concentration of 75 ppm, a very slow reorientation of the molecules is observed.

At a concentration of 500 ppm, bilayer formation starts. However, despite a significant increase in concentration, the associated molecules do not form a second full layer, and rinsing the system with a solvent causes desorption of associated molecules and reversion to the monolayer in the between or end-on orientation (irreversible adsorption bars).

To confirm the observations concerning the orientation of the adsorbed molecules, the thickness of the formed layers was computed using the Navi^TM^ LayerSolver, BioNavis, Tampere, Finland software. The MP-SPR curves representing results for particular orientations of molecules were selected. The following conditions were: 5, 100, 500, and 1000 ppm. The obtained values for both ionic strengths are presented in Table 1. The lysozyme molecule is ellipsoidal, and its dimensions are 4.5 × 3.0 × 3 nm; therefore, a thickness of 3 nm corresponds to the side-on; 4.5 nm to the end-on orientation of molecules in monolayer. In the case of bilayers, it is as follows: 6 nm- side-on; 7.5 nm- side-on *+* end-on; and 9 nm- end-on. The obtained results indicate that for the concentration of 5 ppm for both ionic strengths, molecules are in side-on orientation; at 0.15 M, the almost-complete monolayer is formed. In comparison, at 0.01 M, a twenty-fold increase in concentration to 100 ppm is associated with starting a full side-on-oriented layer and reorientation to the end-on position. At 500 ppm, a complete end-on monolayer is observed and the formation of a side-on bilayer begins at 0.01 M and 0.15 M. The obtained results for molecules’ orientation determined based on the thickness of the lysozyme layers are consistent with those calculated using the RSA model. The change in the lysozyme molecule’s orientation on the sensor surface is confirmed by earlier experimental work [30,36,37,38].

It should be noted that a lower mass characterizes the adsorption of lysozymes on the silica surface rather than on the gold surface [39]. However, Sander et al., modeling based on the quartz crystal microbalance frequency and dissipation, showed that the thickness of lysozyme monolayers adsorbed on silica is about 3 nm, which correlates well with results obtained on the gold surface [40]. According to studies using neutron reflectometry, the thickness of lysozyme film adsorbed on silica with a mass 170 ng/cm^2^ is 30 ± 3 nm, while with a mass equal to 330 ng/cm^2^-50 ± 5 nm [41]. The lysozyme layer’s mass values correspond to reorientation of the molecules from side-on to end-on positions and to side-on bilayer formation, respectively. They are in excellent agreement with the results presented here for lysozyme in the same orientation adsorbed on the gold.

Additionally, the stability of lysozyme adsorption on the gold surface was established by determining the desorption value, which is defined as the difference in the adsorbed mass as a result of supplying a protein solution to the sensor and the mass measured after rinsing the formed layer with a solvent. For both ionic strengths, within the bulk protein concentration range of 5–200 ppm, the desorption does not exceed 15%, proving that the process of adsorption is irreversible in such conditions. At higher concentrations, desorption increases reaching a level not surpassing 30%. The average desorption value for I = 0.01 M is 14.3%, while for I = 0.15 M it is 10.7%, which shows superior adsorption stability at higher ionic strength, and may be due to a more significant association of chloride ions on the gold surface, resulting in the intensified efficiency of adsorption [42,43].

### 2.2. Adsorption of Lysozyme on Gold Surface Monitored by Quartz Crystal Microbalance with Dissipation Monitoring (QCM-D)

**Figure 2:**
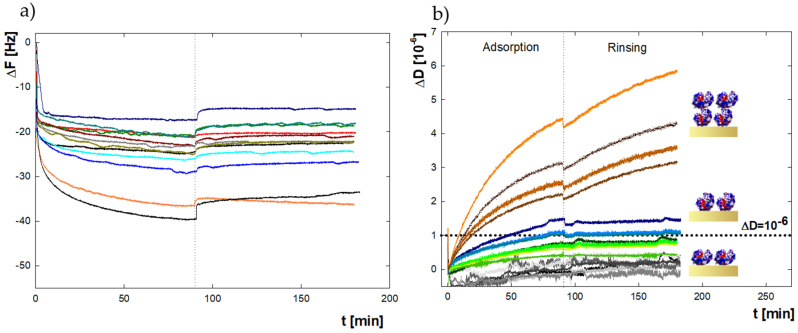
(**a**) Time dependence of the resonance frequency (ΔF) of the QCM-D sensor’s vibrations for bulk concentrations of lysozyme in the range 5–2500 ppm; (**b**) change in the dissipation of energy (ΔD) of the QCM-D sensor as a result of lysozyme adsorption from a bulk protein concentration of 5 ppm (gray), 100 ppm (green), 500 ppm (blue) and 1000 ppm (orange) at I = 0.01 M at pH = 7.5.

Another method that allows studying of the adsorption of proteins on metallic surfaces is QCM-D. It is an acoustic method that provides the possibility not only to record changes in the frequency of sensor’s vibrations depending on the mass of adsorbed molecules on the surface in real-time, but also has a module for monitoring the energy dissipation, which enables the viscoelastic properties of the formed layer to be determined [44,45,46,47,48]. QCM-D experiments were performed with the same sequence and the same conditions as for the MP-SPR method. Figure 2a shows the changes in the sensor’s resonant frequency over time for bulk protein concentration in the range of 5–2500 ppm.

According to the literature, adsorbed layers are characterized by rigid or viscoelastic properties and can be described by the Sauerbrey or Voigt models, respectively [32,49]. The criteria for choosing the appropriate model depend on the value of the achieved dissipation of energy and the distribution of the curves representing frequency overtones. If the dissipation is in the range of 0–1.0 × 10^−6^ and the overtones almost overlap, then the obtained film can be considered rigid. When dissipation exceeds the value of 1.0 × 10^−6^ and the overtones are widely distributed, the layer has viscoelastic properties [49]. The obtained dissipation values for the lysozyme adsorbed on the gold surface for different orientations of molecules, both in the structure of a monolayer and a bilayer, are shown in Figure 2b. For measurements at 5 and 100 ppm, the dissipation is below 1.0 × 10^−6^, so these layers are rigid. It means that films composed of particles in side-on and end-on orientation do not differ significantly in the achieved energy dissipation values. In terms of higher concentrations, we observe an increase in energy dissipation. From a concentration of 500 ppm, where full end-on monolayer is achieved, and bilayer formation begins, the dissipation exceeds the value of 1.0 × 10^−6^, indicating viscoelasticity. A further rise in the concentration causes a sharp increase in dissipation (to the value of 7.2 × 10^−6^ at 2500 ppm), proving the high flexibility of the obtained protein film. The overtone curves for measurements at I = 0.15 M have a similar trend, but they have smaller dissipation values, ranging from 0.1–6.0 × 10^−6^. It may indicate a more rigid compacted structure than the protein layers formed for the same bulk concentration in a solvent with lower salt content.

**Figure 3:**
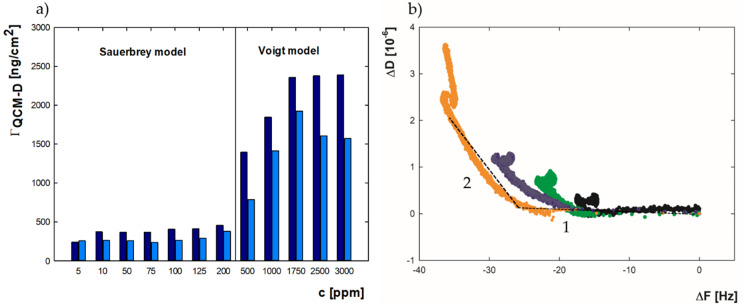
(**a**) The mass of adsorbed lysozyme ($\Gamma_{QCM-D}$) on the gold surface after 90 min of adsorption for I = 0.01 M (navy blue) and for I = 0.15 M (blue) monitored by QCM-D. The Sauerbrey model was used in the concentration range of 5–200 ppm, and the Voigt model from 500 to 2500 ppm (**b**) ΔD as a function of ΔF of the QCM-D sensor as a result of lysozyme adsorption from a bulk protein concentration of 5 ppm (gray), 100 ppm (green), 500 ppm (blue) and 1000 ppm (orange) at I = 0.01 M, pH = 7.5. The numbers indicate the dissipation curve’s parts corresponding to rapid (1) and slow (2) phases of adsorption.

Establishing whether the formed layer is rigid or viscoelastic allows selecting the appropriate model to convert the changes in the frequency of the sensor’s vibration to the mass adsorbed on its surface. In the case of rigid films, the Sauerbrey model- and for the flexible layers, the Voigt model- are used [32,49,50]. Based on the previously obtained information on the dissipation during layer formation, the mass of molecules on the gold surface was calculated, adopting the Sauerbrey model for results in the range of 5–200 ppm, and the Voigt model for higher bulk concentrations. The obtained values for 0.01 M NaCl and 0.15 M NaCl are presented in Figure 3a.

Comparing the results for both ionic strengths shows that, in almost the entire concentration range, greater adsorption efficiency occurs at I = 0.01 M. Importantly, the adsorption efficiency trend for the QCM-D method is different from the MP-SPR method. For QCM-D measurements at 0.01 M in the concentration range of 5–200 ppm, the mass increases gradually from 230 to 450 ng/cm^2^, then at 500 ppm it rises rapidly to 1390 ng/cm^2^ and 1840 ng/cm^2^ for 500 and 1000 ppm, respectively. In the range of 1000–2500 ppm the adsorbed mass is kept constant at 2300 ng/cm^2^. For MP-SPR measurements, the mass gradually increased over the entire concentration range. For I = 0.15 M, the trend for MP-SPR and QCM-D is similar-constant level in the bulk protein concentration range 5–125 ppm ($\Gamma_{QCM-D}\;$= 250 ng/cm^2^), followed by a slight increase in adsorbed mass for 200–500 ppm, and subsequently a sharp increase to the level of adsorbed mass equal to 1500 ng/cm^2^ for 1000–2500 ppm.

The ΔD values as a function of ΔF presented in Figure 3b can be used to describe the differences between the adsorption behavior of lysozyme, dependent on selected conditions. Two fragments can be distinguished in each of the recorded curves. The parts marked as 1 and 2 represent the rapid and slow phases of the process and are characterized by different slopes in the ΔD-ΔF plot [51]. For both parts, the slopes decrease with bulk protein concentration (5–2500 ppm). For the initial stage, the values change slightly. They are in the range from −0.0316 to -0.0590 for I = 0.01 M and from −0.0325 to −0.0695 for I = 0.15 M. For the slow stage, the slopes drop more sharply with concentration to −0.1996 for I = 0.01 M and −0.1279 for I = 0.15 M at 2500 ppm (all of the calculated slopes are presented in Supplementary Materials, Table S1). The higher values for the rapid phase suggest strong interactions between the protein and the surface. Simultaneously, the intensive changes during the second stage are related to the reorganization of molecules inside the layer [52]. The obtained results indicate biphasic kinetics of adsorption involving initial adsorption, and subsequent rearrangements and possible conformational changes in adsorbed molecules, which can be described by the two-state model:

(1) $$A\;\begin{array}{c} k_{a1}\\ ⇆\\ k_{d1} \end{array}\;B\;\begin{array}{c} k_{a2}\\ ⇆\\ k_{d2} \end{array}\;C$$

where states A, B, and C are the gold surface, the surface covered with lysozyme at the initial adsorption, and layer after reorientation of lysozyme molecules, respectively. The two-state kinetics model involves an exchange between a native-like dissolved state and a highly perturbed adsorbed state, which was also previously observed using circular dichroism spectroscopy for lysozyme adsorbed to the fumed silica surface of nanoparticles [53]. For quantitative analysis of the lysozyme adsorption kinetics on the gold, the association constants (k_a_) and dissociation constants (k_d_) for measurements performed by MP-SPR and QCM-D at both ionic strengths were calculated using TraceDrawer, Ridgeview Instruments AB, Uppsala, Sweden software. The OneToTwo kinetic model (with grouped analysis option), which considers rearrangement and conformational changes, was selected for the presented two-step adsorption. A similar approach where structural variations were expected was presented by P. Canoa et al. [54]. The overall constants were calculated based on two equations: $k_{a}=k_{a1}\cdot \;k_{a2}$ and $k_{d}=k_{d1}\cdot k_{d2}$. The obtained overall values are presented in Table 2, while the others are included in the Supplementary Materials (Table S2).

The obtained values show that the association constant is one order of magnitude higher for experiments carried out at 0.15 M. However, under these conditions, the dissociation rate is greater by one order of magnitude for MP-SPR and two orders of magnitude for QCM-D measurements. Summarizing this information, we can conclude that the overall affinity of lysozyme molecules to the gold surface is similar, independent of the selected ionic strength. This was confirmed by MP-SPR experiments, where it was shown that for lower bulk protein concentration, higher efficiency of adsorption occurred at I = 0.15 M, while after achieving the end-on monolayer, the effectiveness of adsorption was greater at I = 0.01 M. Considering the entire range of selected concentrations, the affinity of the molecules will be similar for both ionic strengths; however, it is possible to distinguish between different adsorption tendencies for low and high bulk protein concentrations which have a direct impact on surface coverage. Comparison of the obtained values with literature data based on MP-SPR measurements shows that the association constants achieve similar results to fibrinogen bound to the surface of TiO_2_ nanoparticles, which were equal to 3.71 × 10^6^; 5.37 × 10^6^ and 6.26 × 10^6^ M/s for TiO_2_ modified by alginate, NeutrAvidin, and bare gold, respectively, while the dissociation constants were at the level of 10^−4^ 1/s [54]. For the peptides interacting with gold nanoparticles, the obtained k_a_ values were in the range of 8.8 × 10^2^–7.0 × 10^3^ M/s dependent on the size of nanoparticles, while k_d_ = 6.6 × 10^−4^–3.0 × 10^−2^ 1/s [55]. Analysis of the dissociation constants values acquired in this work, with a simultaneous comparison of the obtained curves to those presented in the mentioned literature, indicates that the dissociation of lysozyme molecules from the gold surface is slight, and the formed layers are very stable.

#### The Hydration and Viscoelastic Properties of Formed Lysozyme Layers

The difference in the obtained values of the adsorbed mass and the tendencies of adsorption for measurements carried out by MP-SPR and QCM-D is directly related to the fact that MP-SPR is an optical method sensitive to changes in the refractive index close to the sensor surface. The QCM-D method considers not only the adsorbed molecules but also the water associated with them [28,32,56]. Therefore, comparing the results obtained by these two methods allows the determination of the water content in the formed layer, which can be calculated using the following formula:

(2) $$\Gamma_{H_{2}O}=\frac{\Gamma_{QCM-D}-\Gamma_{MP-SPR}}{\Gamma_{QCM-D}}\cdot 100\%$$

where $\Gamma_{MP-SPR}$ is the adsorbed mass measured by MP-SPR, $\Gamma_{QCM-D}$ is the adsorbed mass measured by QCM-D, and $\Gamma_{H_{2}O}$ is the percentage of water content in the adsorbed layer.

**Figure 4:**
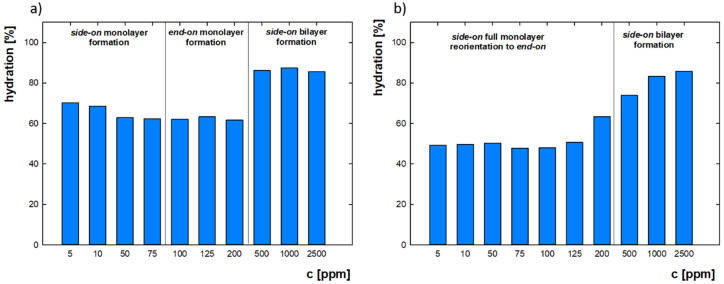
The percentage content of water in the lysozyme layers adsorbed on the gold surface depending on the bulk protein concentration at pH = 7.5 for (**a**) I = 0.01 M; (**b**) I = 0.15 M.

The hydration results depending on the bulk protein concentration for the ionic strength 0.01 M are shown in Figure 4. The bulk protein concentration, which results in lysozyme surface coverage on the gold, significantly affects the obtained layers’ hydration. At 5 ppm, when a full layer in side-on orientation has not been formed, hydration is at the level of 70%. As coverage increases and the monolayer becomes full, the percentage of water in the layer decreases. When full monolayer is reached and the molecules are reoriented to the end-on position, hydration is 62%. A further increase in the concentration and adsorbed mass is associated with the formation of a bilayer and causes a significant increase in hydration to the level of 85%. In the case of measurements in 0.15 M, the trend is different. Firstly, after crossing 5 ppm the monolayer is achieved and molecules started to rearrange to the end-on position. In such conditions, a low hydration level equal to 50% was maintained. A significant change in the lysozyme layer’s water content was recorded at 200 ppm, where the bilayer formation process began, and a rise in hydration was maintained until the end of the selected concentration range, reaching its maximum equal to 85% for 2500 ppm. Presented hydration range values are consistent with previously obtained data, according to which the hydration of protein layers can reach up to about 90% [57]. It was also presented by M. Ouberau et al. that the percentage of water in the lysozyme film adsorbed on silica oxide drops with increasing surface coverage (Θ) from 73% at Θ = 0.25 to about 60% when the monolayer is formed. It was also shown that partial bilayer formation causes an increase in water content, which is in good agreement with results featured in this work [26]. It is worth noting that when the monolayer is completed for the bulk protein concentration, the film is less hydrated for higher ionic strength. The observation is connected with the fact that the presence of NaCl can be responsible for screening the protein charge and enhancing protein-solvent interactions. Whereas in the conditions where a bilayer is formed, the percentage of water is similarly independent of salt concentration, which may indicate that the impact of the screening potential significantly declines in such states. Such an effect may be related to the rearrangement of the water molecules in the protein layer. Previously, it was confirmed that the enhanced presence of ions in a solvent could influence the proportion of different water types in the adsorbed film.

**Figure 5:**
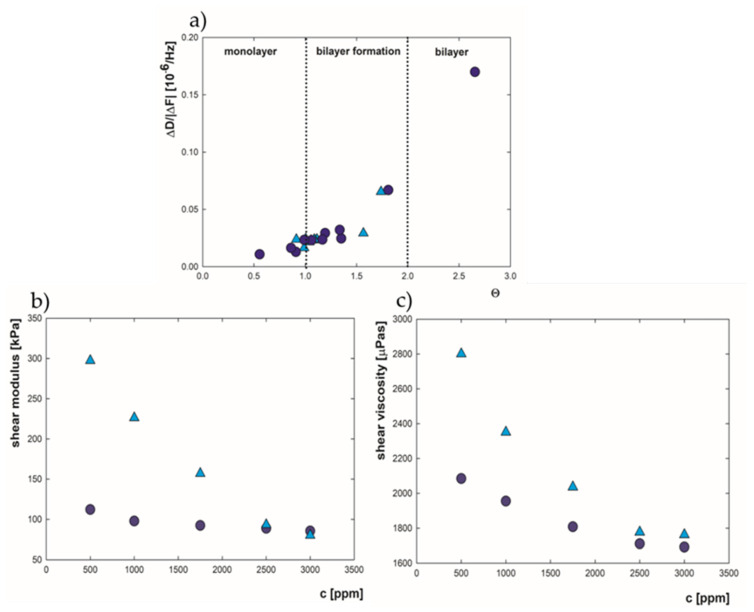
(**a**) The change of dissipation energy per unit of frequency change depending on surface coverage of lysozyme (**b**) shear modulus and (**c**) shear viscosity of adsorbed lysozyme layers on the gold surface depending on the protein bulk concentration at pH = 7.5, I = 0.01 M (●) and I = 0.15 M (▲).

The lysozyme layers obtained under various conditions of concentration and ionic strength differ in structure (partial/full monolayer or bilayer) and hydration. These factors should result in the viscoelastic properties of the obtained layers. To check whether the above-mentioned factors affect the characteristics of adsorbed films, a dependence of energy dissipation per unit of sensor’s vibration changes (ΔD/|ΔF*|*) as a function of surface coverage (Θ) was analyzed and is presented in Figure 5a. The value is considered to be a simple indicator of differences in elasticity properties, and it is used for concluding structural transitions occurring as the adsorbed layer grows in mass [58], because a large increase in the energy dissipation per unit of sensor’s frequency vibration during adsorption is characteristic for less rigid layers. During monolayer formation (Θ < 1.0), low values are achieved, suggesting more stiffness properties. However, the progressive formation of an adlayer is associated with an increase in the obtained ΔD/|ΔF*|* values, which is exceptionally high when the complete bilayer is formed. These results indicate significant changes in the elastic properties occurring during the adsorption of the second lysozyme layer.

The quantity of the changes taking place during adlayer formation was examined by calculating the shear modulus and shear viscosity of adsorbed films using QSense DFind, Biolin Scientific, Espoo, Finland software with the SmartFit model upon the Voigt theory. The fitted viscoelastic properties presented in Figure 5b,c were obtained based on the third to the eleventh overtones of frequency and dissipation curves measured by QCM-D. In the lysozyme concentration range of 500–3000 ppm and 0.01 M NaCl, the shear modulus values decreased from 112 to 83 kPa, while in 0.15 M NaCl the values dropped from 297 to 80 kPa. In other research, for lysozyme adsorbed on methyl, the elasticity was at the level of 900 kPa. However, such a high result is related to the fact that a low water content of 51% was observed under these conditions. In contrast, for the albumin hydrated in 80%, the elasticity modulus was equal to 250 kPa, which corresponds well with those studies [26]. Similar values of shear modulus at the level of 100-kPa were also recorded by I. Sharma and S. Pattanayek for myoglobin, which is of a similar size to lysozyme, in the concentration of 250–1000 ppm dissolved in water and adsorbed on an APDMES surface [59]. According to the presented results, an increase in lysozyme concentration causes a reduction in shear modulus, which is related to more elastic properties during progressive bilayer formation. The shear viscosity of adsorbed lysozyme recorded in this work changed from 2100 to 1690 µPas at I = 0.01 M, and from 2800 to 1750 µPas at I = 0.15 M (Figure 5c). Twice smaller values were observed for more hydrated rich in alpha-amylase salivary layers, characterized by two timeslower shear elasticity [60]. The similar trend of changes in elasticity and viscosity caused by higher bulk protein concentration was also presented for β-lactoglobulin [61]. There were also differences depending on the salt amount in a solvent. The minor changes for lower ionic strength are related to the almost-constant water content at the level of 85% in the formed layers for the selected lysozyme concentration. When the ionic strength is higher, the hydration increases gradually from 72% to 86%, which results in more intense changes in viscoelastic properties. The higher salt content in the solution causes higher layer stabilization through the more substantial contribution of counterion condensation. However, their share is diminished with protein concentration, which, in addition to hydration, affects viscoelasticity changes more significantly. According to which protein layers formed in higher ionic strength are stiffer and more rigid, the trend has been demonstrated for salivary films [60]. Based on the presented results, it can be concluded that the progressive adlayer formation with protein concentration indicates that in lower ionic strength, the film is more homogenous and characterized by similar elastic properties, while in higher NaCl content it is rather heterogeneous—composed of denser, more rigid material near the surface and more flexible near the solution, which is directly associated with the rearrangement of associated water.

### 2.3. Secondary Structure of Adsorbed Lysozyme Measured by Fourier Transform Infrared Spectroscopy (FTIR)

**Figure 6:**
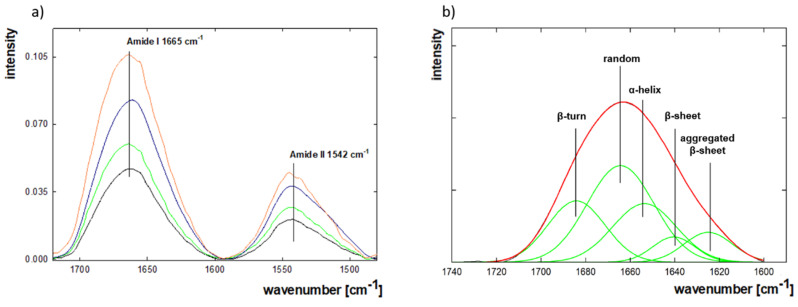
(**a**) FTIR-ATR spectra in the range 1450–1750 cm^−1^ for lysozyme adsorbed on the gold surface depending on the concentration of bulk protein solution (black-5 ppm, green-100 ppm, blue-500 ppm, orange-1000 ppm) at 0.01 M, pH = 7.5; (**b**) a scheme of fitted bands corresponding to the elements of the secondary structure to the area of amide I.

To determine the changes in the adsorbed lysozyme on the gold surface, Fourier-transform infrared spectroscopy (FTIR) with the grazing angle-attenuated total reflection (VariGATR) accessory module analysis was performed. Figure 6a shows the spectra for protein concentrations representing the molecules in different orientations and the form of a monolayer and bilayer (5 ppm-incomplete side-on monolayer, 100 ppm-full side-on monolayer, 500 ppm-complete end-on monolayer, 1000 ppm-bilayer formation) at pH = 7.5 and I = 0.01 M. Analogous experiments were carried out for I = 0.15 M. The positions of the bands corresponding to the particular elements of the secondary structure were determined based on the location of the II-derivative minima of the obtained spectra, and they were assigned to specified structures using literature data: 1626 cm^−1^-β-sheets, 1640 cm^−1^-random structured, 1659 cm^−1^-α-helices, 1666 cm^−1^-3_10_ helices, while the 1684 cm^−1^ corresponds to β-turns, and if there was a band at 1694 cm^−1^ then it was assigned to dehydrated β-turn (Figure 6b) [62]. The percentage content of individual secondary structural elements was calculated based on the area under the bands corresponding to the specified secondary structure element and the area under the spectra of amide I, according to the following equation [63]:

(3) $$\mathrm{II}-\mathrm{structure}\;\mathrm{element}\;\mathrm{component}\;\left(\%\right)=\;\frac{\mathrm{II}-\mathrm{structure}\;{\mathrm{component}}^{\prime }s\;\mathrm{area}}{\mathrm{area}\;\mathrm{of}\;\mathrm{amide}\;I\;\mathrm{band}}$$

Previous studies have indicated that the structure of lysozyme changes due to the interactions between protein molecules and the gold surface. Circular dichroism (CD) measurements for lysozyme in bulk solution at a concentration of 50 ppm in 0.01 M NaCl, pH = 3–10, showed that under such conditions, the protein structure is stable and contains: 35.7% α-helix, 31.1% β-sheet, 32% random structures, and 1.2% β-turn; after the process of adsorption on the gold surface in pH = 6 the α-helix content decreases to the level of 19.2% and β-sheet to 10%, while the total percentage of random and β-turn elements increases to 70.8%. Under more alkylated conditions, the β-sheet content rises in favor of random and β-turn [30]. Similar data were obtained by S.M. Daly et al., who presented a result for one-hour adsorption on the hydrophobic self-assembled monolayer surface of alkanethiols at pH = 7.4. They showed that lysozyme’s structure consists of α-helix-23%, β-sheet-12%, and β-turn/random-65%. Longer adsorption time increased the β-sheet content to 25% [31]. The percentage content of lysozyme’s secondary structure depending on the concentration obtained in this study is presented in Table 3. They correlate very well with the literature data and confirm that, after adsorption on a solid surface, the total content of random + β-turn structures is dominant. It can be noticed that during monolayer formation, due to the increase in the concentration of lysozyme from 5 to 500 ppm at I = 0.01 M, the random + β-turn quantity drops, while the content of β-sheet rises slightly. Previous research showed that β-turn elements affect proteins’ stability by protecting the regions responsible for the active formation and maintenance of the proper structure, or passively allowing its folding. Therefore, it can be assumed that the β-turn protects the protein in the adsorbed state against changes in the direction of aggregation. However, too-strong interactions with the surface or neighboring molecules can have a destructive effect on the structure, causing stress, leading to protein destabilization and aggregation, resulting in higher β-sheet content [64]. The increase in the β-sheet level in the protein structure at I = 0.01 M results from the increasing stress to which the protein molecules are subjected along with the increase in surface coverage in the monolayer. Such a tendency was not observed at I = 0.15 M. It cannot be related to the reorientation of molecules, which occurs for both selected ionic strengths. By increasing the ionic strength, we follow a slightly higher content of α-helix and β-sheet, similar to the structure of the native protein. NaCl stabilizes the lysozyme structure, which is in line with the findings regarding the content of water in the adsorption layer. Bilayer formation is manifested by the increased proportion of α-helix and β-sheet, with a simultaneous decrease in random + β-turn in the whole structure of adsorbed lysozyme. It is related to the fact that molecules forming the sublayer are not directly exposed to the gold surface interactions responsible for the conformational changes. The same effect has been observed in other studies, where protein multilayers were formed [29,31,65].

## 3. Materials and Methods

### 3.1. Materials

Hen egg-white lysozyme (L6876) purchased from Sigma-Aldrich, MO/USA was used for the research. It was used without further purification. Lysozyme solutions were prepared by dissolving protein powder in an aqueous NaCl solution with an ionic strength of 0.01 M or 0.15 M and pH = 7.5. The pH was established by adding small amounts of aqueous solutions of sodium hydroxide and hydrochloric acid. All other chemicals used in the study were purchased from Sigma-Aldrich, St. Louis, MO, USA. The experiments were performed at 298 K.

### 3.2. Methods

#### 3.2.1. Multi-Parametric Surface Plasmon Resonance (MP-SPR)

Lysozyme adsorption measurements on the gold surface were performed using the MP-SPR Navi^TM^ 200 apparatus (BioNavis, Tampere, Finland), a goniometer coupled to a prism (Krechmer mode). The system has two separate channels, and each of them can emit wavelengths of 670 and 785 nm. A peristaltic pump is also included in the system. The MP-SPR apparatus operated in a wide range of scanning angles (40–78°). The immobilization of the particles on the sensor surface covered with a gold layer is monitored by registering changes in the intensity of the set angle or changes in the resonance angle’s value with time. The mass of adsorbed lysozyme particles (*Г_MP-SPR_*) on the gold surface is calculated using the following equation:

(4) $$\Gamma_{\mathrm{MP-SPR}}=\;\frac{\Delta \Theta kd_{LYS}}{\frac{dn}{dc}}$$

where ΔΘ is a change in MP-SPR resonant angle, *k* is a constant for the MP-SPR system, *d_LYS_* is the assumed thickness of the adsorbed lysozyme layer, $\frac{dn}{dc}$ is the refractive index increment; *k × d* ≈ 1.0 × 10^−7^ nm/deg and $\frac{dn}{dc}$ ≈ 0.178 cm^3^/g for *λ* = 670 nm for 0.01 M, while for *λ* = 670 nm for 0.15 M *k × d* ≈ 1.0 × 10^−7^ nm/deg and $\frac{dn}{dc}$ ≈ 0.175 cm^3^/g.

The sequence of MP-SPR measurements was as follows: a baseline for a NaCl solution with a defined ionic strength (0.01 or 0.15 M NaCl) and pH = 7.5 (10 min), then 90 min of lysozyme adsorption with a selected concentration in the range of 5–2500 ppm (0.01 or 0.15 M NaCl, pH = 7.5) and 90 min of rinsing with NaCl solution at the specified ionic strength and pH = 7.5. Based on the MP-SPR results, the thickness of the adsorbed layers was determined using the LayeSolver Navi^TM^ v.1.0.2, BioNavis, Tampere, Finland software. The association, dissociation, and affinity coefficients were calculated using TraceDrawer, Ridgeview Instruments AB, Uppsala, Sweden software.

#### 3.2.2. Quartz Crystal Microbalance with Dissipation Monitoring (QCM-D)

Lysozyme adsorption on the gold surface was also carried out using the QCM-D E1 Q-Sense, Biolin Scientific, Espoo, Finland with a flow module. The measurement sequence and conditions were the same as for MP-SPR. The QCM-D method allows determining both the change in the mass of adsorbed molecules and the adsorption process’s reversibility/irreversibility. During the measurements, two parameters were monitored: the change in the resonance frequency (ΔF) and the change in energy dissipation (ΔD). For thin, homogeneous, and rigid layers, according to the Sauerbrey equation, the resonance frequency is proportional to the mass adsorbed on the surface of the QCM-D sensor (*Г_QCM-D_*). In the case of layers with viscoelastic properties, the adsorbed mass is determined using the Voigt model. This model’s calculations were performed using the QSense DFind, Biolin Scientific, Espoo, Finland program based on the measurement results for 3–11 frequency overtones. The established constant parameters were: the solvent density-998 kg/m^3^ for 0.01 M and 1003 kg/m^3^; and the protein layer density-1113 kg/m^3^ for 0.01 M and 1118 kg/m^3^ for 0.15 M, respectively. The model fit was applied to the time corresponding to lysozyme adsorption. The association, dissociation, and affinity coefficients were calculated using TraceDrawer, Ridgeview Instruments AB, Ridgeview Instruments AB, Uppsala, Sweden software.

#### 3.2.3. Fourier-Transform Infrared Spectroscopy (FTIR)

Fourier-transform infrared spectroscopy (FTIR) measurements were carried out using a Nicolet iS10, Thermo Fisher Scientific, Waltham, MA, USA, FTIR spectrometer with the grazing angle-attenuated total reflection (VariGATR) accessory. They were performed for lysozyme adsorbed on the gold surface. A layer of gold with a thickness of 100 nm was deposited on the glass plate by vapor deposition. Measurements were recorded in the wavenumber range from 700 to 4000 cm^−1^. For each spectrum, 128 scans were averaged with a spectral resolution of 4 cm^−1^. Before each measurement, the background spectrum was recorded, and then it was automatically subtracted from the spectrum of the protein sample. Lysozyme was adsorbed at 4 different concentrations (5 ppm, 100 ppm, 500 ppm, 1000 ppm) in a NaCl solution for two ionic strengths, 0.01 M and 0.15 M, pH = 7.5. The adsorption time was 90 min. After that, the gold surface was rinsed successively for 90 min with NaCl solution with controlled ionic strength and pH. Finally, the surface was rinsed by water at the appropriate pH for 90 min. Before FTIR measurements, the gold surface with the adsorbed protein was dried with a stream of air. For data analysis, Omnic, Thermo Fisher Scientific, Waltham, MA, USA and Origin, OriginLab, Northampton, MA, USA software packages were used.

## 4. Conclusions

In the presented work, the adsorption of hen egg lysozyme on the gold surface was investigated in order to combine the efficiency of the process with the structure of adsorbed layers and their properties. The particles’ orientation and the structure of the obtained layers (monolayer/bilayer) were determined based on measurements using the MP-SPR and QCM-D methods and compared in the RSA model. The analysis of the QCM-D curves showed that two stages could be distinguished in the process of lysozyme adsorption. The first stage mainly determined the process of transporting molecules to the interface, and the second stage was related to the rearrangement of molecules in the adsorption layer. The system association and dissociation constants were determined using the TraceDrawer, Ridgeview Instruments AB, Uppsala, Sweden OneToTwo model, considering the reorientation and conformation changes of the molecule on the adsorption surface. The obtained values reached an order of magnitude of 10^6^–10^8^ for k_a_ and 10^−15^–10^−12^ for k_d_. Parallel measurements of MP-SPR and QCM-D showed that the protein layer’s hydration strictly depends on the degree of coverage of the adsorption surface. During the monolayer formation, the percentage of water in the layer decreased, reaching a minimum of 62% and 50%, respectively, for I = 0.01 M and I = 0.15 M when the monolayer was full. The process of bilayer formation increased the system’s hydration by up to 85% for both ionic strengths. The degree of hydration of the lysozyme layers directly influenced the observed differences in the system’s viscoelastic properties. Low-hydrated layers with a single-layer structure were stiff, while the formation of a bilayer was associated with an increase in the layer’s flexibility. For the full bilayer for I = 0.01 M, the shear elasticity was 83 kPa, and the shear viscosity was 1690 µPass. FTIR experiments also enabled determining of the secondary structure of adsorbed lysozyme. As a result of adsorption on the solid surface, the total content of random structures + β-turn dominated. When filling the monolayer, the random structure + β-turn decreased, while the content of the β-sheet slightly increased. The β-turn elements influenced the stability of proteins and prevented the induction of the aggregation process. Too-strong interactions with the surface or adjacent molecules can have a destructive effect on the structure, stressing the system, leading to destabilization or aggregation of proteins, resulting in higher β-sheet content. The level of the β-sheet with I = 0.01 M resulted from the increasing stress in the monolayer, induced by the rise of the surface coverage. Increasing the ionic strength led to an increase in the content of α-helix and β-sheets to the level observed for the native protein. Therefore, it can be assumed that the presence of NaCl stabilizes the lysozyme structure by changing the structure and density of the layer.

## Figures and Tables

**Table 1:** The thickness of the adsorbed lysozyme layers (d_MP-SPR_) depending on the bulk protein concentration (c) of the solution obtained using the Navi^TM^ LayerSolver, BioNavis, Tampere, Finland software. Additionally, the refractive index (n_MP-SPR_) was determined.

c (ppm)	0.01 M		0.15 M		Orientation
	d_MP-SPR_ (nm)	n_MP-SPR_	d_MP-SPR_ (nm)	n_MP-SPR_	
5	2.78	1.387	2.95	1.388	formation of side-on monolayer
100	3.10	1.390	3.26	1.392	side-on to end-on
500	4.76	1.400	4.83	1.402	end-on to bilayer
1000	5.36	1.412	5.23	1.413	formation of side-on bilayer

**Table 2:** The overall association (k_a_) and dissociation (k_d_) constants were calculated based on fitting the OneToTwo kinetic model using TraceDrawer, Ridgeview Instruments AB, Uppsala, Sweden software to MP-SPR and QCM-D curves measured at pH = 7.5 at I = 0.01 M and I = 0.15 M.

Method, Ionic Strength	Overall Association Constant (k_a_) (M/s)	Overall Dissociation Constant (k_d_) (1/s)
MP-SPR, 0.01 M	1.39 × 10^6^	1.89 × 10^−15^
QCM-D, 0.01 M	2.62 × 10^7^	1.50 × 10^−14^
MP-SPR, 0.15 M	1.45 × 10^7^	1.16 × 10^−14^
QCM-D, 0.15 M	9.29 × 10^8^	1.70 × 10^−12^

**Table 3:** The content of lysozyme secondary structure elements in the adsorbed state depending on the bulk protein concentration at pH = 7.5, I = 0.01 M and I = 0.15 M.

c (ppm)	Orientation	α-Helix (%)		β-Sheet (%)			β-Turn + Random (%)	
		0.01 M	0.15 M		0.01 M	0.15 M	0.01 M	0.15 M
5	formation of side-on monolayer	20.1	21.4	10.2		14.7	69.7	63.9
100	side-on to end-on	19.6	22.1	12.2		14.5	67.8	63.8
500	end-on to bilayer	21.1	22.2	12.4		13.5	66.7	64.1
1000	formation of side-on bilayer	23.4	25.7	17.3		21.8	59.3	52.5

## Data Availability

Not applicable.

## Supplementary Materials

The following are available online at https://www.mdpi.com/1422-0067/22/3/1322/s1. Table S1 presents slopes in the ΔD-ΔF dependence measured by QCM-D: 1st slope represents rapid adsorption and 2nd slope represents slow adsorption for the adsorption. Table S2 presents the 1st (k_a1,_ k_d1_) and 2nd (k_a2,_ k_d2_) steps association and dissociation constants, respectively. They were obtained based on MP-SPR and QCM-D curves measured at pH = 7.5 at I = 0.01 M and I = 0.15 M.

## Abbreviations

MP-SPR	Multi-parametric surface plasmon resonance
I	Ionic strength
QCM-D	Quartz crystal microbalance with dissipation monitoring
*d_MP-SPR_*	Thickness of adsorbed layer obtained based on MP-SPR measurements
*n_MP-SPR_*	Refractive index of adsorbed layer obtained based on MP-SPR measurements
*c*	Concentration of bulk protein solution
$\Gamma_{MP-SPR}$	Adsorbed mass measured by multi-parametric surface plasmon resonance
$\Gamma_{QCM-D}$	Adsorbed mass measured by quartz crystal microbalance with dissipation monitoring
$\Gamma_{H_{2}O}$	Percentage water content in the adsorbed layer
FTIR-ATR	Fourier-transform infrared spectroscopy with the attenuated total reflectance method
