# Supplementary material for: Adsorption and Conformation Behavior of Lysozyme on a Gold Surface Determined by QCM-D, MP-SPR, and FTIR
Source: Int J Mol Sci. 2021 Jan 28;22(3):1322. doi: 10.3390/ijms22031322 (PMC7865459; doi:10.3390/ijms22031322)
Supplement: Supplementary file 1 [file ijms-22-01322-s001.pdf]

Supplementary Materials

Adsorption and Conformation Behavior of Lysozyme on a Gold Surface Determined by QCM-D, MP-SPR, and FTIR.

Table 1. The slopes in the ΔD-ΔF dependence measured by QCM-D: 1st slope represents rapid adsorption and 2nd slope represents slow adsorption for the adsorption in the concentration range 5-2500 ppm, pH = 7.5, at both I = 0.01 M and I = 0.15 M.

c (ppm)	1st slope / rapid adsorption		2nd slope / slow adsorption	
	0.01 M	0.15 M	0.01 M	0.15 M
5	−0.0316	−0.0325	−0.0541	−0.0562
20	−0.0502	−0.0674	−0.0678	−0.0668
100	−0.0566	−0.0694	−0.1099	−0.0820
200	−0.0582	−0.0695	−0.1091	−0.0834
500	−0.0582	−0.0691	−0.1355	−0.1030
1000	−0.0591	−0.0694	−0.1980	−0.1143
2500	−0.0590	−0.0695	−0.1996	−0.1279

Table 2. The 1st (k_{a1}, k_{d1}) and 2nd (k_{a2}, k_{d2}) steps association and dissociation constants, respectively. They were calculated based on fitting the OneToTwo kinectic model using TraceDrawer, BioNavis, Tampere, Finland software to MP-SPR and QCM-D curves measured at pH = 7.5, at I = 0.01 M and I = 0.15 M.

method, ionic strength	1st step association constant (k_{a1}) (M/s)	2nd step association constant (k_{a2}) (M/s)	1st dissociation constant (k_{d1}) (1/s)	2nd dissociation constant (k_{d2}) (1/s)
MP-SPR, 0.01 M	6.97×10^{4}	2.00×10^{1}	7.59×10^{-11}	2.62×10^{-5}
QCM-D, 0.01 M	7.07×10^{4}	2.05×10^{2}	1.57×10^{-9}	7.40×10^{-5}
MP-SPR, 0.15 M	1.54×10^{5}	1.70×10^{2}	4.69×10^{-10}	3.19×10^{-5}
QCM-D, 0.15 M	3.52×10^{5}	2.64×10^{2}	1.05×10^{-7}	1.62×10^{-5}
